# How does prestige bias affect information recall during a pandemic?

**DOI:** 10.1371/journal.pone.0303512

**Published:** 2024-05-16

**Authors:** Edwine Soares de Oliveira, André Luiz Borba do Nascimento, Washington Soares Ferreira Junior, Ulysses Paulino Albuquerque

**Affiliations:** 1 Programa de Pós-Graduação em Etnobiologia e Conservação da Natureza, Universidade Federal Rural de Pernambuco, Recife, Pernambuco, Brasil; 2 Laboratório de Ecologia e Evolução de Sistemas Socioecológicos, Departamento de Botânica, Universidade Federal de Pernambuco, Recife, Pernambuco, Brasil; 3 Universidade Federal do Maranhão, Campus Bacabal, Bacabal, Maranhão, Brasil; 4 Universidade de Pernambuco, Campus Mata Norte, Nazaré da Mata, Pernambuco, Brasil; Federal University of Goias: Universidade Federal de Goias, BRAZIL

## Abstract

The prestige theory of evolution states that our memory has an intrinsic bias to memorize information from someone of prestige. However, the evidence for information recall is mainly focused on content bias. Considering that the prestige bias can be advantageous in selecting information in contexts of uncertainty, this study assessed whether, in the scenario of the COVID-19 pandemic, the prestige bias would be favored over other models that do not possess the prestige spirit characteristics. The study was conducted through an online experiment, where participants were subjected to reading fictitious text, followed by a surprise recollection. Data were analyzed using a generalized linear mixed model, Poisson family, and logistic regression. The results showed that prestige is only prioritized in the recall due to the family model and does not present any difference from the other models tested. However, it influenced the recall of specific information, suggesting its role as a factor of cultural attraction. Furthermore, we observed that trust in science-oriented profiles can influence the recall of information during a health crisis. Finally, this study highlights the complexity of the functioning of the human mind and how several factors can act simultaneously in the recall of information.

## Introduction

Since the human mind can prioritize recalling potentially adaptive information [1], especially in survival contexts, it becomes coherent to believe that it has evolved into an organized and specialized set of biases [2] that act in information selection.

Acquiring information based on the characteristics of the transmitting individual (model-based biases) has been highlighted as one of these selected biases throughout our evolution [3]. This is because selecting information from successful individuals in a given domain (hunting, fishing, medicinal plants) increased the chances of acquiring potentially adaptive information and thus increasing the chances of survival [2]. In this way, selecting information from expert individuals within a given knowledge domain (success bias) is adaptive; however, it is sometimes difficult to evaluate this success directly. Our mind has, therefore, developed shortcuts that help recognize indirect cues that go beyond domain expertise, such as the differential attention that an individual receives [2, 4]. This bias was called prestige bias by Henrich and Gil-White (Prestige Theory of Evolution) [2].

When an individual has knowledge and influence within a particular domain of knowledge, it may indicate that he or she is successful in that area, but not necessarily prestige. This is because prestige emerges from a set of direct and indirect characteristics causes the individual to be seen differently in the population. These characteristics involve personality, appearance, esteem and social status, attention received by others, and influence in a certain area [2, 4, 5] Prestigious people can also be influential beyond their original domain of knowledge (e.g., soccer players influencing haircut models) [4, 6].

Based on this theory, Henrich and Gil-White [2] predicted that information transmitted by prestigious people would be more memorable [2]. Although prestige bias has been tested over the years in contexts of cultural transmission, there are no direct tests in memory itself.

In general, adaptive memory studies have investigated how certain information is prioritized in memory in survival contexts [7–9], which may suggest a content bias. However, there is evidence that, in the transmission process, recall can be influenced not only by the content of the information but also by the prestige of the model that transmits it [10]. This suggests that prestige bias may also operate in information recall.

However, no empirical studies specifically test the effect of prestige bias on recall. The existing evidence involves recall during the transmission process and is still controversial. For example, Jiménez and Mesoudi [9] tested whether information transmitted by prestigious models was better recalled along a transmission chain. The results were not significant for prestige bias. However, this result may have been influenced by the fact that it was not a specific memory experiment [11]. Thus, we cannot be sure how this bias works in the recall of information.

Contexts of uncertainty, where the environment changes very quickly, and it is difficult to predict what may happen, can favor model bias to the detriment of content bias, mainly due to the lack of previous experience that allows more careful analysis of the situation. Information itself [12]. For this reason, memory is no different; in uncertainty, the prestige bias likely acts by making sure information is more memorable and also acts on recall, as Henrich and Gil-White predicted [2].

The COVID-19 pandemic has brought this uncertainty to the whole world, with environmental and social changes and epidemiological information that is often uncertain and changing very quickly [13]. Within this context, the information passed by a model could be much more advantageous than analyzing each piece of information per se [14]. Evidence has already shown that experts in the field [15], politicians [16] and social connections, whether with family members or people close to them [17, 18], can have a strong influence on the way people respond to risks in a pandemic context. Although model bias can generally influence the selection of information [19], according to the theory of prestige evolution [2], depending on the context, this selection can be carried out through indirect clues of success. Even if these do not necessarily have to do with the domain in question, which brings us to prestige models. During the pandemic, mainly due to social isolation measures, social networks played an essential role in people’s lives [20]. Therefore, personalities such as digital influencers (characterized as people with characteristics that can indicate prestige, such as a certain status and social appreciation) gained much notoriety and could be perceived by people as prestigious models.

The pandemic was a scenario of constant change. Initially, even experts in the field were adapting to the situation, and politicians did not know precisely how to act. At the same time, family members shared all kinds of news. Therefore, retaining information from models with evidently prestigious characteristics is more advantageous than from others, such as experts, politicians, and family members without the characteristics considered prestigious in the literature [2, 4, 5].

Thus, our main question is: in a pandemic scenario, does the prestige model lead to greater recall of information than political models, family models, and models with experience in the subject? We hypothesize that a source of social prestige strongly influences the recall of COVID-19-related information. We expect that information related to COVID-19 attributed to someone of prestige (digital influencers) will be remembered more than that attributed to political models with experience in public health and family members.

## Experimental design

The procedures for carrying out this study followed a pre-registered protocol [21]. The few modifications that needed to be made are described below.

### Ethical aspects

The study received approval (No. 5,329,534, on 04/04/2022) from the Human Research Ethics Committee of the Federal Rural University of Pernambuco, as mandated by current Brazilian legislation (Resolution No. 466 of December 12, 2012). All volunteers were asked to complete an online authorization regarding the Informed Consent Form, as guided by Resolution No. 466/12 of the National Health Council. If the volunteer agreed to participate in the study, they were instructed to select the option confirming their participation and understanding of the research proposal after reading the informed consent text. No payment or reward was provided for the participation of each volunteer, as Brazilian legislation does not recommend such remuneration. Data collection commenced on April 06, 2023, and was concluded on May 08, 2023. Only adults aged 18 years or older were recruited for the study.

### Project

We investigated whether people remember more information when attributed to a prestigious source in a pandemic. To do this, we used an online platform without the need for monitoring, which was explicitly designed for this experiment. The experiment was based on a fictional text offered to participants that contained information about using a medication for COVID-19. Four versions of the text were prepared, one for each model to be tested, and each version was composed of the same information; only the model assigned to it changed.

As the experiment was conducted online, researchers did not need to be blinded when analyzing the results. More information about information selection is provided below, along with the detailed procedure of the experiments. All data resulting from the research is included as supplementary information.

### Sample size

Individuals aged 18 or over with internet access were selected to participate in the research. Volunteer recruitment was done through social media platforms (Twitter, Facebook, Instagram, and WhatsApp—Recruitment start: April 06, 2023;

End of recruitment: May 08, 2023). On Instagram, paid traffic was used for about a week to reach more people. After recruitment, people were directed to the online platform (pesquisa.dcfreela.com.br). We performed an *a priori analysis* to determine the sample size and power using the G*Power software [22]. As a basis for the study, we took the effect size (Odds ratio = 1.81) from the study by Bonin et al. [23] as it is the most recent study that brings variables related to memory and uses the same statistical test we had proposed. We calculated a sample size of 327 people with an explanatory power of 95% and z = 1.64. The default for Pr(y = 1|x = 1)H0 in the G*Power software is 0.2 (i.e., a 20% probability of the event occurring if the null hypothesis is true). However, we used 0.1 to obtain an even more reliable sample size for our investigation phenomenon. We consider an α of 0.05, a Power (1-β err prob) of 0.95, R 2; = 0, and normal distribution. Oliveira et al. [21] state the parameters used for this calculation.

#### Data exclusion criteria

Data relating to the following were excluded from the research:

1. Volunteers who participated but were under 18 years old.
2. Volunteers who abandoned the research before the end of the experiment.
3. Volunteers who requested the exclusion of their data from the research.
4. Volunteers who did not perform the attention/distraction test activity.
5. Volunteers who did not complete the recall stage and reached the end of the experiment.
6. Volunteers who have accessed the platform more than once can access texts using different model manipulations.
7. Volunteers who needed help understanding what was being asked in the recall stage have yet to respond to what was requested.

### Sample demographics

A total of 582 people accessed our platform and participated in the research. However, after the exclusion criteria, our final sample consisted of 317 participants, all residents of Brazil, and distributed across 22 states (federative units). The percentage distributions of gender identity, sexual orientation, education, political inclination, and income are found in S1 File.

### Selection of information used

As information about COVID-19 was in wide circulation among the population for more than two years, we did not use data on symptoms and real forms of treatment and prevention, as there is a risk of bias. Therefore, we create fictional information appropriate to the context. To select our prestige model, we considered the characteristics described in the scale developed by Berl et al. [18] to assess how people attribute prestige. These characteristics are based on social status, esteem, influence, and knowledge of a particular subject. Due to isolation measures during the COVID-19 pandemic, social networks played an essential role in communication [20]. From this, personalities such as digital influencers gained much notoriety, constituting an ideal proxy for the prestige model in this study. In this case, digital influencers receive significant attention (the more followers, the greater their engagement and popularity), have a prominent social status, experience within their niche, and influence beyond that niche.

To decide on the other models used (compared with the prestige model), we selected a model that knew the public health topic but no prestige to assess whether prestige is more influential than experience in this scenario. We used a study by Hornsey et al. [24] for the other models, stating that psychological roots are behind our attitudes toward accepting or rejecting scientific information. Six roots are then described: ideologies, vested interests, conspiratorial worldviews, fears and phobias, personal identity expression, and social needs. Conceptually, these roots are distinct but may overlap [24]. Thus, we select models who may have behaviors and attitudes that reflect the overlap of these roots, such as politicians, who share the same ideologies and interests; family members, who along with previous roots, may also have the same world views but may also validate opinions related to personal fears and phobias. Furthermore, empirical studies have shown how politicians’ opinions are considered when making decisions during a pandemic [16]. Recent studies also reveal that family care is a human priority [18], so it is plausible that people within families can act as role models.

So that the selected models did not contradict the ideologies of each participant, we ensured that they were not named and were on the political positions and personal tastes of each reader, highlighting this in the texts (Table 1).

**Table 1:** Models used in preparing the text to carry out the information recall experiments about COVID-19.

Models	
Prestige	A digital influencer with many followers and considerable influence that the participant follows and admires
Political	Politicians aligned with the participant’s political position and who they would vote for
Experience/knowledge	Doctor who worked on the frontline against COVID-19
Parental	Family member (who the participant considers to be someone close to them)

### Preparation of texts

The texts contained a brief contextualization of the pandemic scenario, followed by a private opinion on the use of the drug Postex in the treatment of COVID-19 attributed to one of the models. When attributing opinions to models, we described the characteristics that conferred prestige or not. This way, we could determine whether the prestige model influences information retention. The stories had approximately the same number of words, ranging from 123 to 129.

All texts had the same structure: first, the pandemic context and then a model with an opinion on medicine use. The number of central propositions (critical information points in a narrative) referring to each text is also the same (Table 2).

**Table 2:** Central propositions used in the story texts for the COVID-19 information recall experiments.

Central propositions	
Proposition 1	Contextualization of the pandemic period
Proposition 2	Model characterization
Proposition 3	Support for the use of medication
Proposition 4	Decrease the number of deaths
Proposition 5	Decrease the number of hospitalizations
Proposition 6	Claim that use is safe
Proposition 7	Success rates exceed failure rates
Proposition 8	Postex must be used

All texts were randomized, so they had the same probability of being assigned to participants.

### Data collection procedure

#### Recall experiment

The experiment was carried out using a protocol adapted from the study by Silva et al. [25], which involves offering reading material to a participant, immediately followed by a distraction activity, and then performing a surprise recall test of the information that was read.

The participant could access the Free and Informed Consent Form (TCLE) by clicking the research link. After participating, the volunteer went to the next page, which contained a form to fill out their data (name, sexual orientation, gender identity, date of birth, education, occupation, region, and state where they live, income, political inclination, and religion). In this questionnaire, some specific points needed to be modified from the version published in the registered study protocol. The issue regarding sex was divided into sexual orientation and gender in order to address all existing expressions and types. Therefore, in addition to the options to be marked, there was a brief description of each and a box with the option "other" since there is a possibility that we did not cover them all. The income question was also better explained, making it clear that it consisted of individual income. The value was updated according to the Brazilian minimum wage during data collection.

After completing the questionnaire, participants were directed to the next page, where the following message was: “*Now we ask you to read the text below*. *Please read carefully as some questions will be asked later”*. Below the message was one of the texts previously randomized by the platform. After reading the text, they were directed to a brief attention test, a distraction activity. The test consisted of identifying specific figures. Each page had a time limit of 2 minutes, which was essential to ensure standardization in the time spent by participants.

Finally, the participants underwent the recall test, in which they were instructed to write the respective text as accurately as possible. The instructions were: “*Now write what you can remember about the text you read previously*. *Be as accurate as possible*, *and do not worry if you cannot remember all the information*. *You have 10 minutes to complete this activity*.” The delimitation of time at this stage had the objective of establishing standardization of the maximum time that each participant would have to perform the activity and was based on experiments that have already used a free recall test, as well as ours [26] At the end of the activity, each person was directed to a self-report stage of multiple-choice questions, with open options to leave examples. The questions were: [i] Who do you trust to seek information about COVID-19? (Participants could choose more than one option, with the alternatives being health professionals, family members, friends, and politicians, among other everyday personalities); [ii] What is your most significant source of information about COVID-19? (They could choose more than one option, and the alternatives ranged from online media to physical media, such as newspapers and magazines); [iii] Have you been affected by COVID-19? (The options here were yes or no; if yes, they were asked to what extent they were affected); [iv] Have any members of your family been affected by COVID-19? (The options were the same as the previous question); [v] Have you been vaccinated against COVID-19? (The objective of this step was to observe whether self-reported sources of confidence influenced information recall). After completion, there was a message of recognition and thanks, and the participant left the platform (all questionnaires and figures relating to the data collection procedure are available at doi.org/10.1371/journal.pone.0281991).

### Data analysis

To analyze the recall of information attributed to each model, we considered the central propositions recalled by participants in each of the conditions presented in the texts. Central propositions are the key points of information in a narrative. Each text contained eight propositions, including the social context and the model to which information was attributed. Each proposition was assigned a binary classification to signal recall. As participants were instructed that they would not need to remember all the text details, it was considered presence if the proposition’s meaning remained, even with other words or sentence construction. For example, the original text contained the proposition “he *is categorical in stating that postex must be used*," we considered that the participant remembered when he wrote, " *he strongly recommends the use of the medication*."

To investigate whether prestige bias affects the amount of information recalled, we performed a generalized linear mixed model, Poisson family, and to investigate the recall of each specific proposition, we performed a multilevel logistic regression, both tests considering the participant as a random effect. Moreover, as a fixed effect, the model type (digital influencer, medical, political, and family). To test the validity of the statistical models, they were compared with a null model (which only considered the effect of grouping by participants) using the chi-square (X^2^) test for model adjustments through the ANOVA function and the maximum estimate likelihood.

The same analysis was conducted to analyze the self-report information and the total number of propositions recalled. To exclude the effect of content on recall, we performed an X^2^ test to evaluate the most recalled propositions and, from there, observe whether they were linked to any specific content. All tests were carried out in the R environment using the lme4 package [27] (see S1–S5 Files).

The statistical models used in this study assume the independence of the observations and the linearity of the relationships between the variables. Although the tests performed were carefully selected to meet these assumptions, we recognize that other relationship forms between the variables may exist and were not explored in this analysis. It is also important to highlight that the tests are based on data collected in a specific context, a self-report of participants in a controlled environment, which may limit generalization to other populations or real-world situations. However, this methodology is the most appropriate approach to the research proposed in the study.

## Results

### Predictive model–prestige bias vs. models with no defined prestige

The number of recalled propositions attributed to the prestige model (influencer) differed significantly only from the family model (Table 3). In summary, the information attributed to the prestige model (influencer) was remembered more than the family model. It showed no differences between the medical and political models, which allows us to refute our hypothesis.

**Table 3:** The generalized linear mixed model (Poisson family) was stabilized based on the influencer (prestige bias) and generated to identify whether the model bias influences the number of propositions recalled.

Fixed effect	Coefficient (standard error)	Z value	Pr (>|z|)
Intercept	1.237 (0.061)	20.032	<2e-16 ***
Family	-0.183 (0.088)	-2.058	0.0396 *
Doctor	-0.031 (0.088)	-0.352	0.7247
Political	-0.094 (0.088)	-1.066	0.2863
**Random effect**	Variance	Standard deviation	
Participants	0	0	
AIC	1208.7		

*p < 0.05

#### Recall of specific propositions

Subsequently, we carried out predictive models to recall each text’s central proposition. To achieve this, each statistical model was stabilized by influencer (prestige bias). For *the general context of the pandemic*, the influencer was not more relevant in the memory than the other models (family, medical, and political) (Table 4). For the *characteristics of the model*, the influencer proved to be more relevant in recall when compared only with the family model. At the same time, the doctor and politician showed no difference in recall of the proposition (Table 4). Regarding *support for medication* use, the influencer proved to be more relevant in remembering the proposition than the family and medical model. In contrast, with the political model, there was no significant difference in recall (Table 4).

**Table 4:** Multilevel logistic regression models are generated to understand which model bias significantly affects the recall of specific information. Each column concerns the model generated for specific propositions.

	Context	Model features _	Support for the use	Decrease in deaths	Decrease in hospitalizations		Safe use	Success rates exceed failure rates	Postex must be used
Fixed effect	Coefficient (standard error)	Coefficient (standard error)	Coefficient (standard error)	Coefficient (standard error)		Coefficient (standard error)	Coefficient (standard error)	Coefficient (standard error)	Coefficient (standard error)
Intercept	0.83 (0.25)*	2.28 (0.39)*	1.32 (0.28)*	-0.77 (0.24)*		-1.67 (0.31)*	-2.00 (0.35)*	-1.57 (3.046e-01)*	-0.89 (0.25)*
Influencer vs Family	-0.38 (0.33)	-1.63 (0.45)*	-0.96 (0.35)*	0.26 (0.33)		0.62 (0.40)	-0.76 (0.58)	-1.43 (5.959e-01)*	-0.09 (0.35)
Influencer vs Doctor	-0.06 (0.35)	-0.88 (0.49)	-0.78 (0.36)*	0.11 (0.34)		0.90 (0.39)*	-0.44 (0.55)	1.96 (4.308e-01)	0.12 (0.35)
Influencer vs Politico	-0.58 (0.33)	0.05 (0.56)	-0.15 (0.38)	0.04 (0.34)		-0.27 (0.46)	-0.50 (0.55)	-4.87 (4.669e-01)	-0.13 (0.35)
**Random effect**	Variance (standard deviation)	Variance (standard deviation)	Variance (standard deviation)	Variance (standard deviation)		Variance (standard deviation)	Variance (standard deviation)	Variance (standard deviation)	Variance (standard deviation)
Participants	1.6e-07	2.5e-11	0	4.08e-08		4.067e-08	1.6e-07	4e-08	1.6e-07
	(4e-04)	(5e-06)	(0)	(0.000202)		(0.0002017)	(4e-04)	(2e-04)	4e-04
AIC	421.2	288.8	391.1	415.9		328.6	187.9	237.6	387.6

*Regarding the number of deaths* reduced, the influencer was no longer relevant to recall other models (family, medical, and political) (Table 4). As for *reducing the number of hospitalizations*, the medical model was more relevant in the recall due to the influencer, while the other models did not show significant differences (Table 4).

To *affirm safe use*, the influencer also did not prove to be more relevant about any other model (family, medical, and political) (Table 4). Regarding *success rates exceeding failures*, the influencer was more relevant than the family model and showed no difference from the other models (Table 4). Finally, concerning the proposition *that posts should be used*, the need to be more was irrelevant compared to other models (Table 4). These results indicate that, although prestige bias does not influence the total amount of information recalled, it does influence the recall of some specific information.

**Fig 1:**
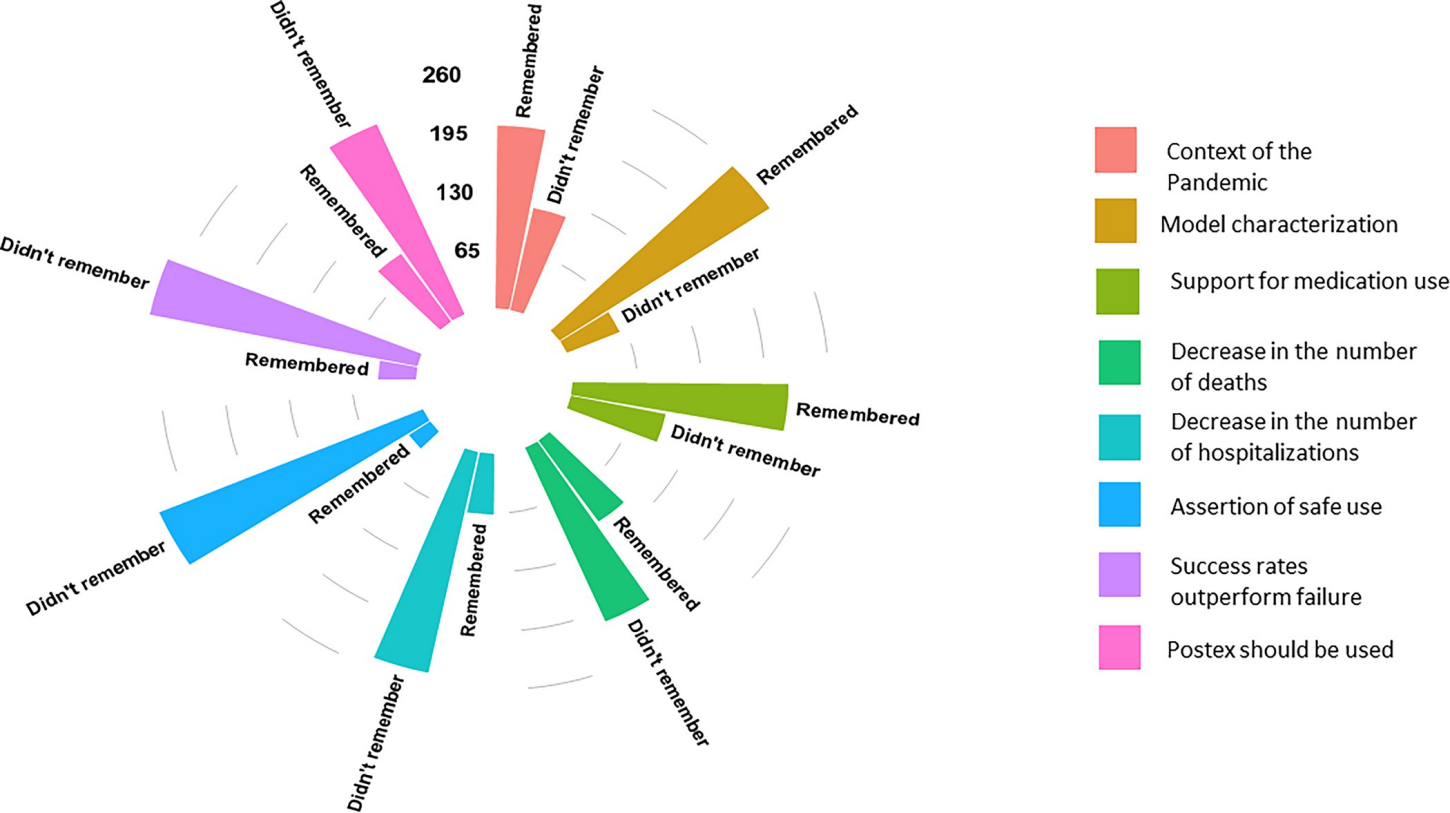
Graphical representation of the experiment participants’ most and least remembered memories.

When analyzing the number of memories each proposition in the text obtained, we observed that the first three were remembered more (with a positive effect). In contrast, people remembered the last five less (with a negative effect) (Fig 1).

#### Participants self-report

After the recall test, participants indicated who they trusted to receive information about COVID-19, the sources from which they received news about it, whether or not they were affected by the disease, including whether any family member was also affected, and whether the participant had been vaccinated against the disease. In the options about trust and source of information, they were asked to leave real examples of these people and sources.

We carried out a predictive model to evaluate whether the options listed about whom to trust to receive information influenced the amount of information recalled. The complete model (model carried out with all tested variables) showed three variables close to a significant result, and based on this, we removed the other variables to adjust the model (S2 File). When performing this adjustment, the three variables (official institutions, digital influencers, and scientists) showed a significant positive effect on the amount of information recalled (AIC of the full model: 1209 | AIC of the adjusted model: 1203) (S3 File). It is worth highlighting that the examples written about the digital influencers and the scientists the participants used to trust were all Brazilian scientific communicators who were in the spotlight throughout the pandemic. The institutions were described as universities, research agencies, and health units.

The model carried out to analyze whether the source from which the information came was related to the total information recalled did not present any significant results. Likewise, the model that evaluated the relationship between being affected by COVID-19 and vaccination, with recall also did not present significant results (S4 and S5 Files).

## Discussion

We expected that information attributed to a model with specific characteristics that confer prestige (in this study, a digital influencer) would be remembered more than the same information attributed to models who did not have these explicit characteristics, such as politicians, doctors with experience in public health and family members. Contrary to what we predicted, our results showed that prestige bias only influenced the amount of information recalled to the detriment of the family model, showing no significant differences between the medical and political models. Therefore, our hypothesis was refuted.

Some explanatory paths can be taken to understand this result. First, there is the possibility that the other models (experts and politicians) also represent prestigious models for the research participants, and, therefore, the recall of information attributed to them did not differ from that of the digital influencer. However, we have not tested this and cannot safely conclude that these models also represented prestigious personalities.

Another explanatory path would be to assume that, contrary to what Henrich and Gil White’s hypothesis [2] predicts, perhaps there is no innate bias in memory to remember better information attributed to someone of prestige. This statement gains support when we observe previous studies, which, although they did not carry out a specific memory study, also provided evidence of the absence of prestige in memory [11]. Unlike our study, which was carried out in a pandemic context (where obtaining information can increase the chances of survival), the results of these authors [9] addressed a context with less adaptive appeal. This strengthens the argument that prestige is not a relevant bias in memory, whether in everyday contexts or health crises.

Our findings, however, concern short-term memory, and as this is a cross-section of this aspect of memory, we cannot say with certainty that the absence of prestige is an innate bias in all memory. There is evidence that human cognition has a neural apparatus that allows the recognition of social position (one of the primary cues of prestige), from the innate perception of indicative signs to the ability to learn these [28]. The role of prestige becomes even more complex, as evidence indicates its effect on associative memory [29], while it does not seem to influence the linking of events in episodic memory [30]. In other words, the effect of prestige is related to the type of memory to which the information is related, not influencing short-term memory, as in our study, and episodic memory, but influencing associative memories. More investigations need to be conducted to determine whether this occurs and possible explanations.

Finally, there is the possibility that prestige acts according to specific domains in pandemic contexts, which differs from what we tested in our experiment. The model chosen in our study was prestige between domains (a digital influencer, influential in a given area, widely followed, and with great reach). There is robust evidence that when choosing a model of prestige, people tend to follow a hierarchy; that is, domain-specific prestige (that, in addition to success, the individual exhibits the cues of prestige within a specific domain) tends to be the most preferred, followed by general domain, and finally cross-domain prestige [31]. That may be why its influence on recall did not stand out over models from specific domains, such as politicians and experts, but was prioritized due to the family model. We suggest that future investigations test the prediction of Henrich and Gil-White [2] based on domain-specific prestige.

Additionally, we found that, although prestige does not influence the recall of the number of propositions remembered, about other models tested, it was relevant for the recall of some specific propositions. In this sense, Berl et al. [8] point out prestige bias can interact with content bias. If this were the case with our results, it would help to understand the significant effect of prestige on specific information, mainly because there is already strong evidence of the influence of content on recall [7, 32]. However, this was not the case in our case because not all of the most recalled information (which could indicate a possible content effect) were those that had a significant effect of prestige bias. So, what could explain the relevance of prestige bias in recalling some information? There is a complementary phenomenon to the selection of information called cultural attraction [33]. Cultural attraction is the probability that certain items are favored within cultural chains to the detriment of others, which can affect the frequency of these items within a population [34, 35]. The cultural attractor would be a particular cultural trait with greater frequency and stability within the population, and this occurs because certain factors "draw attention" to that specific trait. These cultural attraction factors can be psychological or environmental [35].

Since prestige was only significant for some information, in our experiment, it may have acted as a cultural attraction factor for the recall of certain information, which would be the *characteristic propositions of the model*, *supporting the use of the medication*. *Moreover*, *success rates exceed failure rates*. At the same time, the contents of these propositions themselves would be the cultural attractors. These specific contents provide a general overview of what may be relevant when receiving information about possible treatment amid a health crisis. For example, the description of who was passing on the information (referring to the characteristics of the model), that is, whether it could be a reliable model or not, that person’s opinion regarding the treatment (referring to their support for its use) and evidence concrete assessment of its effectiveness (referring to success rates). For this reason, these propositions were more attractive than the others that only reinforced these arguments and were not as attractive. Analyzing prestige biases as possible factors of cultural attraction can elucidate a series of studies in which their effect has yet to be defined entirely [10, 36].

When observing the direct influence of transmission models on the recall of specific propositions, we observed that in those in which the prestige bias proved relevant, it was to the detriment of the medical and family models. This may reveal that its role as an attraction factor is so vital that it overcomes mastery experience and familiarity with other individuals, biases that also exist within context biases [19]. However, it did not show a significant difference with the political model; its effects on recall were similar. This suggests that even without the evident characteristics conferring prestige on a given model, the politician can still exert influence to the point of resembling the prestige model and influencing the information people adopt [16, 37].

Our results also indicated that the profile of who participants trusted to receive information during the pandemic influenced the amount of information recalled. Among the personalities indicated as trustworthy in receiving news, the profile focused on science stood out, including research institutions, scientists, and scientific communicators. During the pandemic, the importance of scientific communication in disseminating information about COVID-19 was reinforced [13, 38, 39]. Examples of social networks, such as Twitter, showed that information from experts was more valuable than that from public authorities [15]. Our results, together with this evidence, strengthen the argument that in periods of crisis, science-oriented profiles play a crucial role in the dissemination of information. This can be investigated in a more concrete way, based on experiments that directly investigate the role of models with these profiles in the recall of information and how this information is shared in the population. Once the direct role of science-oriented profiles in communication is verified, these people can be allies in the strategic communication of truthful information about facing future public health crises. However, factors such as the source of information, whether or not the person had been affected by COVID-19, and whether they had been vaccinated did not show significant effects.

The lack of effects of information sources differs from evidence showing news sources (such as newspapers and digital media) as predictors of beliefs related to COVID-19 [40]. Our evidence suggests that the transmitter model is more relevant for information recall than the location where the information is being conveyed.

Another relevant point is that direct experience with the disease also did not affect recall, even though empirical evidence indicates that prior experience is essential in recall [26]. This may have been due to people’s indirect experience [41], as COVID-19 is a recent and widely spread disease, and information about it was in constant circulation. Therefore, regardless of whether it was hit or not, everyone was immersed in this context, and perhaps that is why there was no difference in the amount of information remembered. A similar phenomenon may have occurred for the effects of vaccination since practically all research participants were vaccinated. However, we emphasize that since the influence of indirect experience on recall was not directly tested in our study, it is necessary to test this in the future for more robust conclusions.

## Limitations

Although our experiment was carried out based on a pandemic scenario, the timing of data collection happened after isolation measures had been relaxed and close to the end of the pandemic. For this reason, our results may not have been influenced by crucial aspects related to pandemic psychology, which in turn may have influenced the model biases acting during the critical period of the COVID-19 pandemic.

Another point is that if, on the one hand, recruiting participants through virtual means did not allow a greater and more varied reach of people, on the other hand, it restricted our sample to people with internet access. For this reason, we need to exercise caution when generalizing our results to the entire population.

When analyzing the most remembered propositions, it was found that people remembered the first three. At the same time, from the fourth onwards, their recall decreased considerably, demonstrating a possible methodological effect of our work and not an effect of the information content in itself.

There is evidence that the order of information presented can affect which information is remembered most [42]. This was done, for example, in studies that used texts with different points of view [36]. However, as it is a text without any controversial arguments, we do not balance the order in which the information is presented. It is essential that future studies that seek to investigate the recall of information from texts pay attention to this and provide versions with the presentation of information in different orders.

We recognize that these limitations may have influenced part of the results found. However, these limitations did not make the studied phenomenon unfeasible since we could clearly visualize the results we proposed to study despite them.

## Conclusions

In the context of COVID-19, prestige was not a determining factor in the amount of information remembered by people, which does not entirely exclude the role it can play in memory but suggests that its performance may be related to specific domains. This suggests that the action of biases in recall is even more complex than we imagined since different factors may be acting together.

Furthermore, the results indicated that certain information is more likely to be remembered within the same context than others. This may indicate the existence of other criteria that need to be addressed in this work, influencing this prioritization of information. Identifying these criteria is essential to establish the best communication strategies during public health crises.

Another interesting point was the influence of science-oriented profiles on information recall. This indicates that scientific communication can be essential in disseminating correct information during periods of health crises. And if future studies, in fact, demonstrate in a more targeted way how this can happen, people with this profile can be allies in the elaboration and dissemination of more strategic communication within health crises. Finally, this study highlights the complexity of the functioning of the human mind and opens gaps for investigations into which domains and contexts prestige can act.

## Supporting information

S1 File Socioeconomic characterization of the sample. (DOCX)

S2 File A generalized linear mixed model (Poisson family), generated to identify if self-reported confidence in receiving information about COVID-19, influences the number of propositions recalled. (DOCX)

S3 File Best fit of the generalized linear mixed model (Poisson family) for self-reported confidence in receiving information about COVID-19 and its relationship with the amount of information recalled. (DOCX)

S4 File Generalized linear mixed model (Poisson family) for self-reported sources from which participants usually received information about COVID-19 and their relationship with the amount of information recalled. (DOCX)

S5 File Generalized linear mixed model (Poisson family) for self-reporting about being affected by COVID-19 and vaccination and its relationship with the amount of information recalled. (DOCX)
