# Supplementary material for: How does prestige bias affect information recall during a pandemic?
Source: PLoS One. 2024 May 16;19(5):e0303512. doi: 10.1371/journal.pone.0303512 (PMC11098362; doi:10.1371/journal.pone.0303512)
Supplement: S1 File — (DOCX) [file pone.0303512.s001.docx]

| **Socioeconomic data** | **%** |  | **%** |  | **%** |  | **%** |  | **%** |
| --- | --- | --- | --- | --- | --- | --- | --- | --- | --- |
| ***gender identity*** |  | ***sexual orientation*** |  | ***Education*** |  | ***Political leaning*** |  | **Income** |  |
| Cis man | 34.06 | Asexual | 1.26 | Elementary school (incomplete) | 4.73 | center | 7.88 | < 1 minimum wage | 25.55 |
| Cis woman | 63.40 | Bisexual | 11.35 | Complete primary education) | 22.39 | Right | 10.72 | 1 to 2 minimum wages | 27.76 |
| Non-binary | 0.63 | Heterosexual | 75.07 | Incomplete high school) | 0.31 | Left | 61.51 | 2 to 3 minimum wages | 15.77 |
| Other | 0.94 | Homosexual | 9.77 | Complete high school) | 6.30 | none | 19.87 | > 3 minimum wages | 30.91 |
|  |  | Pansexual | 1.57 | Graduation (incomplete) | 17.03 |  |  |  |  |
|  |  | Other | 0.94 | Bachelor's degree (complete) | 27.44 |  |  |  |  |
|  |  |  |  | Master's degree (incomplete) | 4.41 |  |  |  |  |
|  |  |  |  | Master's degree (complete) | 5.04 |  |  |  |  |
|  |  |  |  | Doctorate (incomplete) | 6.30 |  |  |  |  |
|  |  |  |  | Doctorate (complete) | 5.99 |  |  |  |  |

Supplementary material 1. Socioeconomic characterization of the sample
