# Supplementary material for: How does prestige bias affect information recall during a pandemic?
Source: PLoS One. 2024 May 16;19(5):e0303512. doi: 10.1371/journal.pone.0303512 (PMC11098362; doi:10.1371/journal.pone.0303512)
Supplement: S2 File — (DOCX) [file pone.0303512.s002.docx]

Supplementary material 2. A generalized linear mixed model (Poisson family), generated to identify self-reported confidence in receiving information about COVID-19, influences the number of propositions recalled.

| Fixed effect | Coefficient (standard error) | Z value | Pr (>\|z\|) |
| --- | --- | --- | --- |
| Intercept | 0.94 (0.12) * | 7.33 | 2.29e-13 *** |
| Health professionals _ | 0.15 (0.12) | 1.28 | 0.1976 |
| Institutions | 0.20 (0.09) * | 2.09 | 0.0365 * |
| Family | 0.01 (0.08) | 0.14 | 0.8831 |
| Friends | -0.10 (0.10) | -0.96 | 0.3335 |
| Religious leader | 0.03 (0.41) | 0.09 | 0.9263 |
| Political | -0.22 (0.15) | -1.38 | 0.1647 |
| digital influencer | 0.23 (0.10) * | 2.26 | 0.0233 * |
| Scientists | 0.13 (0.07) | 1.84 | 0.0650 |
| Other | 0.10 (0.16) | 0.62 | 0.5296 |
| **Random effect** | Variance (standard deviation) |  |  |
| Participants | 0  (0) |  |  |
| AIC | 1209.0 |  |  |

*p < 0.05
