# Supplementary material for: How does prestige bias affect information recall during a pandemic?
Source: PLoS One. 2024 May 16;19(5):e0303512. doi: 10.1371/journal.pone.0303512 (PMC11098362; doi:10.1371/journal.pone.0303512)
Supplement: S3 File — (DOCX) [file pone.0303512.s003.docx]

Supplementary material 3. Best fit of the generalized linear mixed model (Poisson family) for self-reported confidence in receiving information about COVID-19 and its relationship with the amount of information recalled.

| Fixed effect | Coefficient (standard error) | Z value | Pr (>\|z\|) |
| --- | --- | --- | --- |
| Intercept | 1.07 (0.04) * | 25.41 | <2e-16 *** |
| **Institutions** | 0.21 (0.09) * | 2.29 | 0.0219 * |
| **digital influencer** | 0.20 (0.10) * | 2.04 | 0.0409 * |
| **Scientists** | 0.14 (0.07) * | 1.96 | 0.0494 * |
| **Random effect** | Variance (standard deviation) |  |  |
| Participants | 0  (0) |  |  |
| AIC | 1203.0 |  |  |

*p < 0.05
