# Supplementary material for: How does prestige bias affect information recall during a pandemic?
Source: PLoS One. 2024 May 16;19(5):e0303512. doi: 10.1371/journal.pone.0303512 (PMC11098362; doi:10.1371/journal.pone.0303512)
Supplement: S4 File — (DOCX) [file pone.0303512.s004.docx]

Supplementary material 4. Generalized linear mixed model (Poisson family) for self-reported sources from which participants usually received information about COVID-19 and their relationship with the amount of information recalled.

| Fixed effect | Coefficient (standard error) | Z value | Pr (>\|z\|) |
| --- | --- | --- | --- |
| Intercept | 0.95 (0.09) * | 10.32 | <2e-16*** |
| **Newspapers** | 0.12 (0.08) | 1.53 | 0.125 |
| **videos** | 0.11 (0.07) | 1.46 | 0.143 |
| **Social media** | 0.12 (0.06) | 1.88 | 0.059 |
| **Others** | 0.10 (0.07) | 1.45 | 0.146 |
| **Random effect** | Variance (standard deviation) |  |  |
| Participants | 0  (0) | - |  |
| AIC | 1207.8 |  |  |

*p < 0.05
