# Supplementary material for: How does prestige bias affect information recall during a pandemic?
Source: PLoS One. 2024 May 16;19(5):e0303512. doi: 10.1371/journal.pone.0303512 (PMC11098362; doi:10.1371/journal.pone.0303512)
Supplement: S5 File — (DOCX) [file pone.0303512.s005.docx]

Supplementary material 5. Generalized linear mixed model (Poisson family) for self-reporting about being affected by COVID-19 and vaccination and its relationship with the amount of information recalled.

| Fixed effect | Coefficient | Standard error | Z value | Pr (>\|z\|) |
| --- | --- | --- | --- | --- |
| Intercept | 0.907 | 0.262 | 3,458 | 0.0005 |
| **Reached by covid** | -0.006 | 0.065 | -0.097 | 0.9230 |
| **Affected family member** | -0.019 | 0.118 | -0.165 | 0.8685 |
| **Vaccination** | 0.279 | 0.244 | 1,141 | 0.2538 |
| **Random effect** | Variance (standard deviation) |  |  |  |
| Participants | 0  (0) |  |  |  |
| AIC | 1207.8 |  |  |  |

*p < 0.05
